# Network pharmacology integrated with molecular docking technology to reveal the potential mechanism of Shuganfang against drug-induced liver injury

**DOI:** 10.1097/MD.0000000000036349

**Published:** 2023-12-01

**Authors:** Ying Wang, Xueying Chen, Yan Wang, Hong Zhong, Liqin Liu, Yang Ye

**Affiliations:** a Zhejiang Hospital of Integrated Traditional Chinese and Western Medicine, Hangzhou, China; b The Second Affiliated Hospital, College of Medicine, Zhejiang University, Hangzhou, China.

**Keywords:** drug-induced liver injury, molecular docking, network pharmacology, Shuganfang

## Abstract

This study aimed to investigate the active composition and mechanism of the Shuganfang (SGF) in treating drug-induced liver injury (DILI) using network pharmacology and molecular docking. The potential active ingredients and targets of SGF were obtained from the Traditional Chinese Medicine Systems Pharmacology Database (TCMSP) database. DILI-related targets were queried from various databases including GEO, GeneCards, OMIM, NCBI, and DisGeNET. The STRING database was used to establish a protein-protein interaction (PPI) network. DAVID was utilized for conducting gene ontology (GO) function enrichment and Kyoto encyclopedia of genes and genomes (KEGG) pathway enrichment analyses. The data visualization and analysis of herb-ingredient-target and disease-pathway-target-ingredient networks were conducted using Cytoscape software (version 3.7.2). PyMoL and AutoDock software was used to select the best binding target for molecular docking. A total of 177 active ingredients,126 targets and 10112 disease targets were obtained, including 122 intersection targets. The identified potential active ingredients consisted of quercetin, kaempferol, luteolin, tanshinone IIa, nobiletin, isorhamnetin, beta-sitosterol and naringenin. The core targets implicated in the study were IL6, estrogen receptor 1 (ESR1), hypoxia-inducible factor alpha subunit 1 (HIF1A), MYC and vascular endothelial growth factor A (VEGFA). KEGG analysis revealed that the treatment of DILI with SGF mainly acted through apoptosis, the PI3K-Akt signaling pathway, and the tumor necrosis factor (TNF) signaling pathway. Furthermore, the binding affinities between the potential ingredients and the core targets were subsequently confirmed through molecular docking experiments. The findings indicated that the docking outcomes remained consistent and demonstrated a favorable capacity for binding. SGF exerts a therapeutic effect on DILI through multiple active ingredients, multiple targets and multiple pathways. Our findings contribute to a positive investigation and establish a theoretical basis for further extensive exploration of SGF as a potential treatment for DILI in future research.

## 1. Introduction

Drug-induced liver injury (DILI) pertains to the aberrant results of liver function tests attributed to the medicinal compounds, pharmaceuticals, dietary supplements, and botanical substances. The estimated occurrence of DILI is between 14 and 19 instances per 100,000 people.^[1,2]^ In most western nations, DILI constitutes over 50% of cases of acute liver failures.^[3]^ DILI is associated with severe adverse outcomes such as hepatitis, liver fibrosis, liver failure, and mortality. DILI can present with a hepatocellular, cholestatic or mixed pattern of disease.^[4,5]^ These various forms of liver injury may necessitate distinct clinical approaches and treatment strategies.^[6]^ Despite the sensitivity of currently used liver parameters, the exact pathophysiology and specific therapies for DILI remain unclear.^[7]^ Therefore, there is an urgent need to develop novel medications for DILI that possess both safety and efficacy.

In recent years, traditional Chinese medicine (TCM) has growing interest due to its safety and multifaceted impacts. Consequently, TCM has gained significant recognition as a viable approach for preventing and treating contemporary diseases.^[8]^ Notably, the utilization of TCM in the treatment of DILI has yielded unique benefits and significant breakthroughs exemplified by the successful application of Hugan tablets,^[9]^ *Schisandrae Chinensis Fructus*.^[10]^ Shuganfang (SGF), a classical TCM formula, show significant effects on treating DILI. It is mainly comprised of *Radix Bupleuri* (Chaihu, CH), *Sedi Herba* (Chuipencao, CPC), *Paeoniae Radix Alba* (Baishao, BS), *Schisandrae Chinensis Fructus* (Wuweizi, WWZ), *Atractylodes Macrocephala Koidz* (Baizhu, BZ), *Aurantii Fructus* (Zhiqiao, ZQ), *Radix Salviae* (Danshen, DS), *licorice* (Gancao, GC). Clinical investigations have demonstrated that SGF has significant therapeutic effect and few adverse in the treatment of DILI.^[11]^ However, there is still a need to elucidate the intricate mechanism of SGF in liver protection.

Network pharmacology is a multidisciplinary interdisciplinary that has arisen under the umbrella of big data in biomedicine and artificial intelligence.^[12]^ Network pharmacology offers a novel approach to uncover the underlying mechanisms of complex systems, encompassing multiple components such as TCM. In this approach, database screening, computer simulation, and information mining techniques are employed to acquire essential targets, pivotal pathways, and mechanisms of action, thereby facilitating a comprehensive elucidation of the intricate biological mechanisms underlying complex diseases and the molecular-level effects of pharmaceutical interventions.^[13]^ TCM exhibits its therapeutic effects by modulating various biological processes through the interaction of multiple targets and bioactive components. In recent years, network pharmacology has emerged as a valuable tool for investigating the intricate mechanisms underlying the efficacy of TCM in complex systems.^[14–16]^

**Figure 1.**
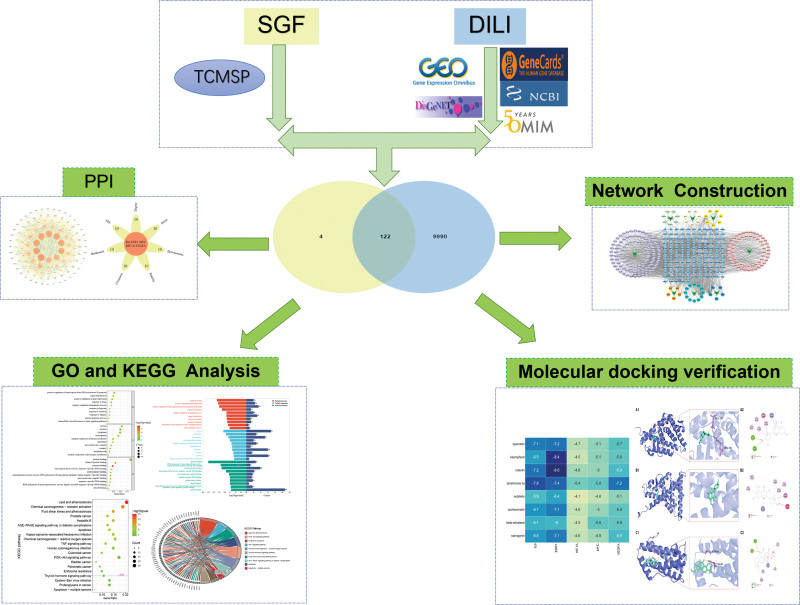
The flowchart of the research.

In this particular study, network pharmacology and molecular docking analysis were utilized to predict the bioactive ingredients, potential molecular targets, and signaling pathways associated with the hepatoprotective effects of SGF against DILI. The results obtained from this research significantly contribute to a more comprehensive comprehension of the pharmacodynamics and mechanisms of action of SGF in the management of DILI. The study workflow is illustrated in Figure 1.

## 2. Materials and methods

### 2.1. Collection of active ingredients and targets of SGF

The active ingredient information of SGF (CH, CPC, BS, WWZ, BZ, ZQ, DS, GC) were obtained from Traditional Chinese Medicine Systems Pharmacology Database (TCMSP, https://old.tcmsp-e.com/tcmsp.php).^[16]^ The TCMSP platform provided crucial information on the constituents of commonly used Chinese herbal medicines, as well as relevant parameters related to their absorption, distribution, metabolism, and excretion (ADME). Subsequently, the active ingredients that satisfied the criteria of oral bioavailability ≥ 30% and drug-likeness ≥ 0.18 were selected for subsequent target prediction.^[17,18]^ In addition, the corresponding targets of these active ingredients in SGF were obtained and compiled from the TCMSP database. The obtained targets name was converted to the official gene symbol using the UniProt protein database (https://www.uniprot.org).^[19]^ The active ingredients and corresponding targets were uploaded to the Cytoscape 3.7.2 to construct the herb-ingredient-target network.^[20]^

### 2.2. Screening targets related to DILI

The differentially expressed genes (DEGs) related to DILI were identified from Gene Expression Omnibus (GEO, www.ncbi.nlm.nih.gov/geo/),^[21]^ Series: GSE54255 (5 healthy controls and 5 DILI). The “limma” package was used for identifying DEGs with logFC > 1 and *P* < .05. The R packages ggplot2 and heatmap were utilized to visualize the DEGs results. In addition, the key words“drug-induced liver injury” were searched in GeneCards (http://www.genecards.org),^[22]^ OMIM database (http://www.omim.org/),^[23]^ NCBI database (https://www.ncbi.nlm.nih.gov/gds/),^[24]^ and DisGeNET database (http://www.disgenet.org/)^[25]^ for the targets related to DILI. Finally, the DILI-related targets library was established by eliminating duplicate targets. Targets from SGF and DILI-related targets were intersected to determine intersections using Venny 2.1.0.

### 2.3. Protein-protein interaction (PPI) network construction and analysis

We imported the intersection targets to the STRING database to acquire the information pertaining to protein-protein interactions.^[26]^ The selection criteria were restricted to “Homo sapiens” with the confidence index ≥ 0.4. Subsequently, PPI data were visualized with Cytoscape3.7.2 to perform a topological and cluster analysis. The key targets in PPI network were estimated using the topological parameters degree centrality more than twice the median. To further screen for hub genes in the PPI network, the Cytoscape plug-in and CytoHubba were utilized.^[27,28]^ The ranking of CytoHubba included 8 methods: MNC, Degree, EPC, Betweenness, Closeness, Radiality, BottleNeck, and Stress analysis.^[29]^

### 2.4. Gene ontology (GO) and Kyoto encyclopedia of genes and genomes (KEGG) analysis

The GO and KEGG pathway enrichment analyses were carried out using the Database for Annotation, Visualization and Integrated Discovery (DAVID version 6.8, https://david.ncifcrf.gov/).^[30]^ The analysis results were filtered based on the following criteria: *P* < .05. Molecular function (MF), cellular component (CC), and biological process (BP) are all included in the GO enrichment analysis. R software was used to visualize the top 10 GO and top 20 KEGG pathway results with the lowest *P* value. The top 20 pathways and their corresponding targets and ingredients were sorted out and the disease-pathway-target-ingredient network was constructed using Cytoscape3.7.2.

### 2.5. Molecular docking analysis

Further molecular docking verification was conducted on the top 8 ingredients identified from the herb-ingredient-target and the 6 hub targets obtained from the PPI network. AutoDock Vina 4.2 and AutoDockTools 1.5.6 were employed for conducting molecular docking analysis.^[31]^ Firstly, the structure of the ingredients was downloaded from PubChem database.^[32]^ The crystal structure of the protein targets was acquired from the RCSB Protein Data Bank (http://www.rcsb.org/).^[33]^ In this specific process, all proteins were dehydrated, the original ligands were extracted, and stored separately. Subsequently, the ligands and proteins were imported into AutoDockTools 1.5.6 in PDBQT format, and a docking grid box was constructed. Finally, the docking and identification of the optimal construct were performed using AutoDock Vina 4.2. PyMOL and Discovery Studio 2020 software were utilized for visualization of ingredients and proteins molecular docking.

## 3. Results

### 3.1. Bioactive ingredients and targets of SGF

**Figure 2.**
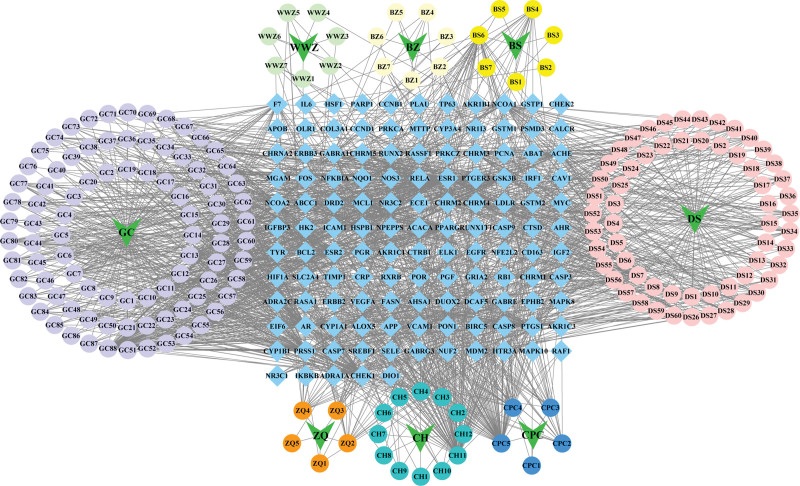
The network of herb–compounds–targets.

There are 12 ingredients for Chaihu, 5 ingredients for Chuipencao, 7 ingredients for Wuweizi, 7 ingredients for Baishao, 7 ingredients for Baizhu, 60 ingredients for Dansheni, 5 ingredients for Zhiqiao, and 88 ingredients for Gancao. A comprehensive collection of 177 active ingredients of SGF was obtained subsequent to the elimination of 14 duplicated ingredients. Through an extensive search of the TCMSP database, we successfully identified 126 targets that corresponded to the aforementioned 177 SGF ingredients. The herb-ingredient-target network, which encompassed the 177 active ingredients and the 126 targets, consisted of 302 nodes and 2201 edges (Fig. 2). In this network, the active ingredients were represented by circles, the therapeutic targets were denoted by blue diamonds, and the herbs were symbolized by green arrows. Further analysis of the network topology revealed that the top 10 ingredients with the highest degree were quercetin, kaempferol, luteolin, tanshinone IIa, nobiletin, isorhamnetin, beta-sitosterol, naringenin, 7-Methoxy-2-methyl isoflavone, isocryptotanshi. The aforementioned ingredients potentially serve as the primary active constituents of SGF for the management of DILI. The results indicate that SGF may exert the therapeutic effects in treating DILI through multiple ingredients and multiple targets.

### 3.2. Prediction of the targets of action of DILI

**Figure 3.**
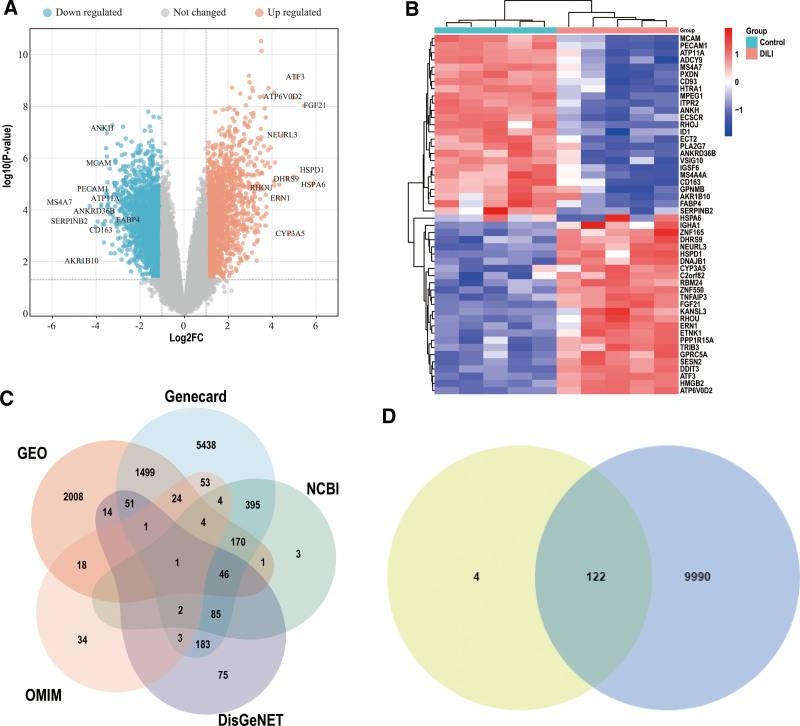
Screening of SGF-DILI common targets. (A) Volcano map of DEGs related to DILI. (B) Heat map of the expression patterns of the top 25 DEGs. (C) The Venn diagram of DILI therapeutic targets in 5 disease databases. (D) Venn diagram of the SGF-DILI common targets. DEGs = differentially expressed genes, DILI = drug-induced liver injury, SGF = Shuganfang.

Through the examination of the GSE54255 datasets in GEO, we identified 3717 DEGs in DILI. Among these DEGs, 657 genes exhibited up-regulation, while 2060 genes displayed down-regulation. The volcano plot illustrating the distribution of the DEGs is depicted in Figure 3A, whereas Figure 3B presents the expression patterns of the top 25 DEGs. In addition, by utilizing GeneCards, NCBI, OMIM, and DisGeNET databases and removing any duplicate entries, we were able to obtain a comprehensive list of 10112 DILI-related disease targets, as depicted in Figure 3C. In Figure 3D, 122 intersections were identified between the 126 SGF ingredient targets and the 10112 DILI-related disease targets using the Venny2.1 online mapping tool.

### 3.3. PPI network analysis

**Figure 4.**
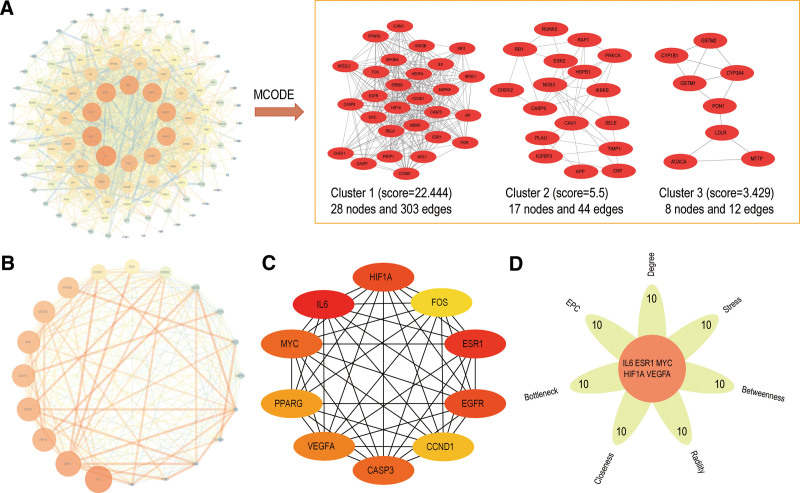
The process of topological screening for the PPI network. (A) MCODE analysis of the common targets. (B) The top 20 targets in the PPI network. (C) MCC analysis of the common targets. (D) The core 5 targets in the PPI network. PPI = protein-protein interaction.

The PPI network of the 122 intersection targets was visually depicted in Figure 4A, comprising 118 nodes and 1182 edge. The nodes in the network were differentiated by size and color, indicating varying degrees. MCODE was used to analyze gene clusters and 3 clusters of targets were generated. The 20 targets with degree values more than twice the median value are shown in Figure 4B. The greater the value of degree, the larger these nodes and brighter the color. To further identify key targets, the network was subjected to analysis using the CytoHubba plug-in (Fig. 4C). Eight algorithms were applied and 5 key targets were obtained: interleukin 6 (IL-6), estrogen receptor 1 (ESR1), hypoxia-inducible factor alpha subunit 1 (HIF1A), MYC, vascular endothelial growth factor A (VEGFA) (Fig. 4D).

### 3.4. GO functional analysis

**Figure 5.**
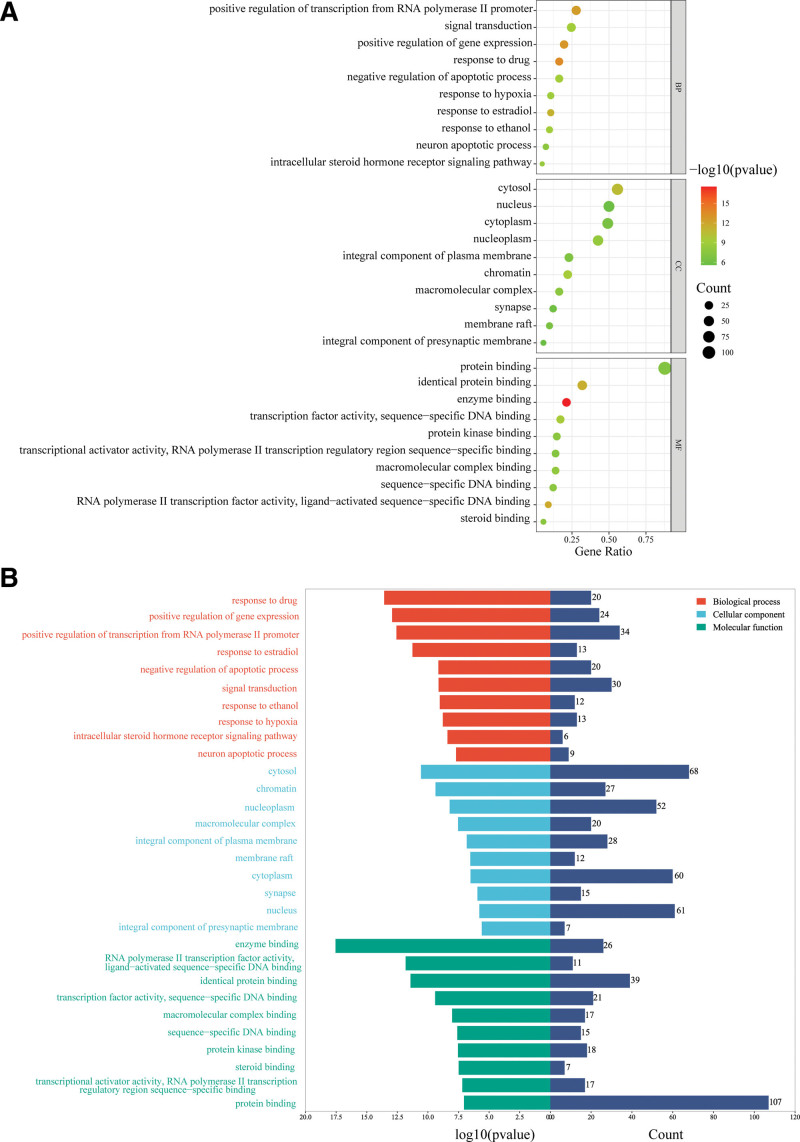
GO enrichment analysis results of biological processes, cell composition, and molecular function annotation. GO = gene ontology.

To further investigate the diverse mechanisms of SGF in the treatment of DILI, 3 levels of GO analysis were done: BP, MF, and CC. BP terms were primarily encompassed positive regulation of signal transduction, gene expression, response to drug, negative regulation of apoptotic process, positive regulation of gene expression, negative regulation of apoptotic process, signal transduction, positive regulation of gene expression, intracellular steroid hormone receptor signaling pathway. MF terms were primarily focused on enzyme binding, ligand-activated sequence-specific DNA binding, identical protein binding, transcription factor activity, RNA polymerase II transcription factor activity, sequence-specific DNA binding, macromolecular complex binding, sequence-specific DNA binding, protein kinase binding, steroid binding. The CC terms were mainly related to the cytosol, chromatin, nucleoplasm, macromolecular complex, plasma membrane. Figure 5A and B illustrates these results.

### 3.5. KEGG pathway enrichment analysis

**Figure 6.**
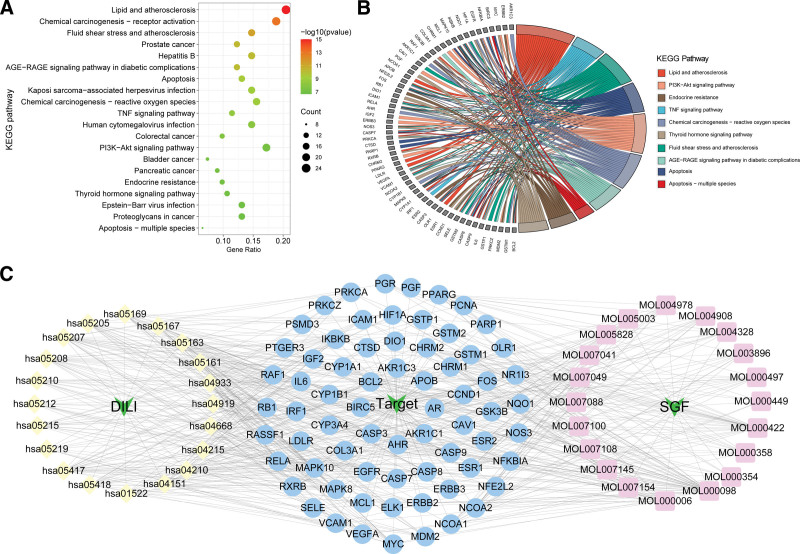
Results of KEGG enrichment analysis and key pathway network construction. (A) The bubble chart of the KEGG enrichment analysis. (B) A chord diagram of the top 10 KEGG pathways. (C) The disease-pathway-targets-ingredients network. KEGG = Kyoto encyclopedia of genes and genomes.

**Figure 7.**
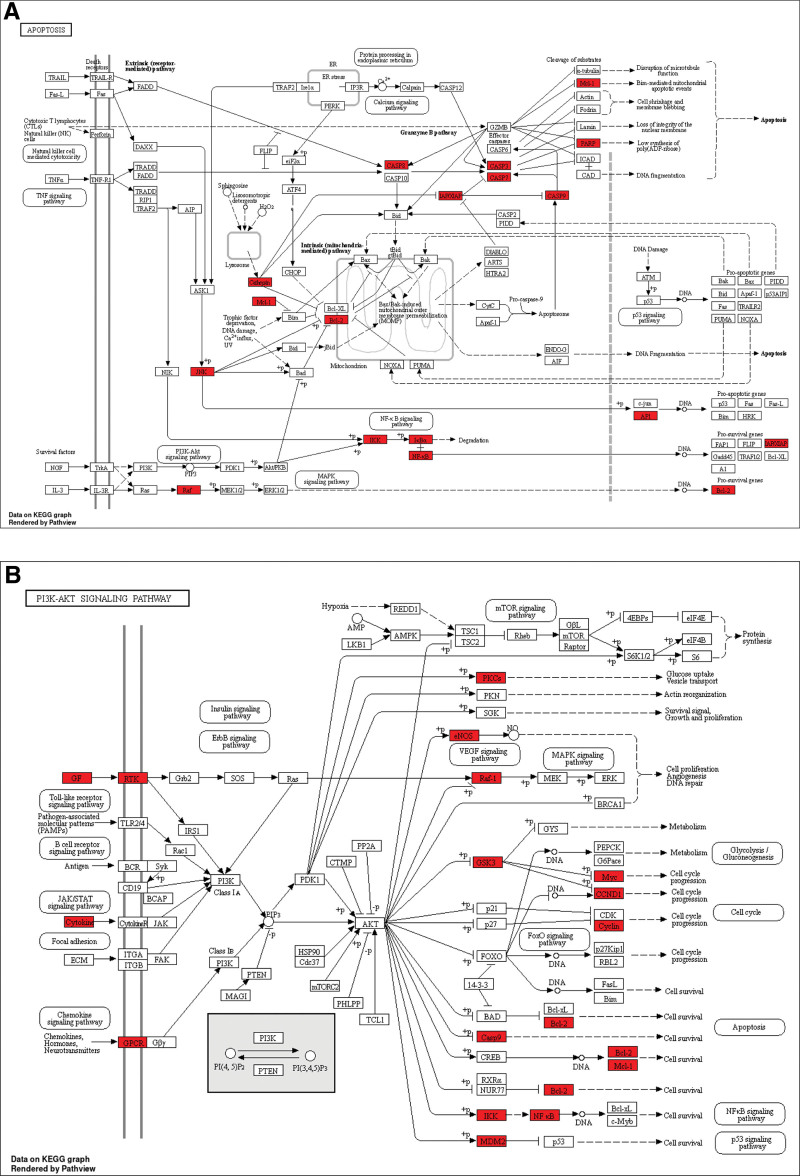
KEGG signaling pathway diagram. (A) Apoptosis signaling pathway. (B) The PI3K/AKT signaling pathway. KEGG = Kyoto encyclopedia of genes and genomes, PI3K = phosphoinositide 3-kinase.

According to KEGG enrichment results, 116 pathways were significantly enriched with the significance level used was *P* < .05. The selection of the top 20 highly enriched pathways was conducted by filtering gene ratios and P values, as depicted in Figure 6A. Our objective was to investigate the potential biological mechanism of DILI, thus pathway terms associated with other diseases and diverse functional categories were excluded. A chord diagram created according to the relationships between enriched pathways and targets utilizing R software (Fig. 6B). In order to visually illustrate the therapeutic mechanism of SGF in treating DILI, we chose the top 20 pathways to constructed a network comprising 113 nodes and 646 edges, representing the disease-pathway-targets-ingredients relationship. This network was created using Cytoscape 3.7.2 (Fig. 6C). KEGG enrichment analysis analysis revealed that the mechanisms of SGF of anti-DILI are mainly concentrated in the PI3K-Akt signaling pathway, apoptosis, the chemical carcinogenesis-receptor activation, and the tumor necrosis factor (TNF) signaling pathway. The apoptosis and PI3K-Akt signaling pathway were visualized using the “pathview” package in R (Fig. 7A and B).

### 3.6. Molecular docking

**Figure 8.**
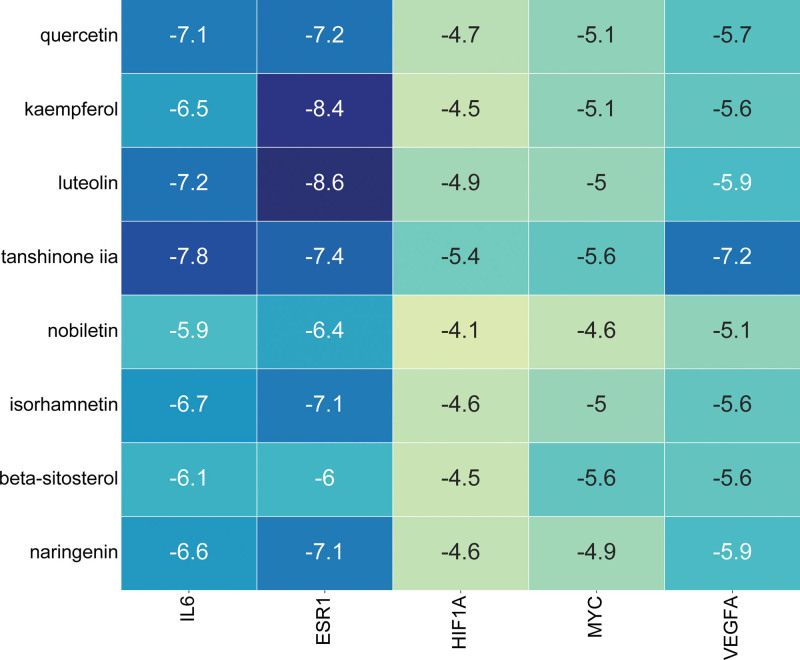
The heat map shows the binding free energy of molecular docking.

**Figure 9.**
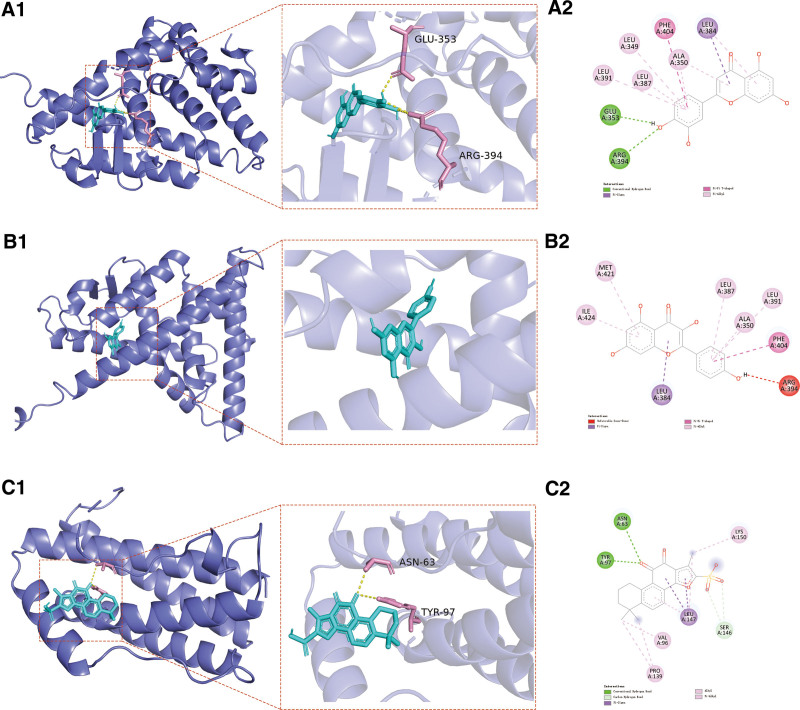
Molecular docking results: (A) Interactions between ESR1 and luteolin. (B) Interactions between ESR1 and kaempferol. (C) Interactions between IL6 and tanshinone iia. ESR1 = estrogen receptor 1.

In order to enhance the credibility of the forecasts derived from network pharmacology, molecular docking analyses were conducted for the top 8 active components including quercetin, kaempferol, luteolin, tanshinone iia, nobiletin, isorhamnetin, beta-sitosterol and naringenin with the top 5 core targets (IL6, ESR1, HIF1A, MYC, and VEGFA). Figure 8 illustrates the molecular docking outcomes, wherein the intensity of color signifies the strength of binding activity between the components and targets. The outcomes of molecular docking analysis unveiled that the highest scoring target proteins and compound ligands were ESR1 and luteolin (docking score = −8.6), ESR1 and kaempferol (docking score = −8.4), IL6 and tanshinone iia (docking score = −7.8), respectively. The simulation diagrams of molecular docking are visualized in Figure 9.

## 4. Discussion

Drug-induced liver injury is a prevalent clinical issue that poses a substantial risk of morbidity and mortality. Nevertheless, there is currently a lack of effective treatments for DILI.^[34,35]^ The identification of interventions capable of treating DILI and impeding its progression into liver failure would greatly benefit public health.^[36]^ At present, TCM has been scientifically proven to exhibit notable therapeutic efficacy for liver diseases through its multi-component and multi-target pharmacological effects.^[37]^ Shuganfang (SGF), a TCM formulation, has been extensively practiced in clinical settings for the treatment of liver diseases over a considerable period of time. Nevertheless, a thorough understanding of the active constituents and molecular mechanisms underlying the effects of SGF remains elusive.

Network pharmacology, an interdisciplinary field integrating molecular biology, genetics, and computer science, is employed to gather, analyze, extract, and apply biological information.^[8,38]^ This approach enhances the ability to predict and categorize diverse therapeutic targets and interactions. Molecular docking-based virtual drug screening has become an integral aspect of structural biology. This study effectively employed network pharmacology and molecular docking methodologies to conduct a comparative analysis of mechanisms, with the objective of elucidating and identifying the constituents and hepatoprotective mechanism of SGF.

The active ingredients of SGF were identified through a comprehensive search of the TCMSP database. Subsequently, a herb-ingredient-target network was constructed and analyzed, resulting in the identification of the top 10 active ingredients, which include kaempferol, luteolin, quercetin, tanshinone IIa, nobiletin, isorhamnetin, beta-sitosterol, naringenin, 7-Methoxy-2-methyl isoflavone, and isocryptotanshi. Research has demonstrated that quercetin possesses the capacity to enhance the expression of Nrf2/HO-1, thereby ameliorating acute liver injury in rats.^[39]^ Kaempferol has been found to possess the ability to inhibit hepatocyte apoptosis, thereby providing protection against liver failure in mice. This protective effect is achieved through the regulation of the ER stress-Grp78-CHOP signaling pathway.^[40]^ It has been demonstrated that luteolin exhibits antioxidative, anti-inflammatory, and anti-endoplasmic reticulum stress properties in mitigating acetaminophen-induced liver injury in mice.^[41]^ Furthermore, prior administration of tanshinone IIA has has been demonstrated to effectively safeguard the liver against APAP-induced hepatic injury through the activation of the Nrf2 pathway.^[42]^ Isorhamnetin has been found to effectively mitigate liver injury induced by acetaminophen through the reduction of oxidative stress, inflammation, and pyroptosis.^[43]^

The PPI network was subjected to topology analysis, resulting in the identification of key targets such as IL6, ESR1, HIF1A, MYC, and VEGFA. IL-6, IL-6, a multifunctional cytokine, is implicated in inflammatory responses and cellular immunity, and it plays a pivotal role in the restoration of hepatic function following liver injury.^[44]^ Recent studies indicates that IL-6 can provide protection against acute liver injury (AILI) by directly signaling through IL-6R on hepatocytes,^[45]^ The IL-6/IL-6R signaling pathway activates the STAT3 pathway, leading to the inactivation of caspases and a reduction in reactive oxygen species.^[46]^ ESR1 has been extensively characterized in the human liver.^[47]^ Abnormal expressions of ESR1 in the liver have been implicated in the stimulation of hepatocyte injury and may serve as inducers or promoters of liver disease.^[48]^ HIF-1α, the master regulator of oxygen homeostasis, plays a crucial role in maintaining oxygen balance in response to hypoxia, inflammation, and oxidative stress.^[49]^ In APAP-induced liver injury, HIF-1α exhibits dual functions, promoting early damage and providing protection later in the pathogenesis.^[50]^ VEGFA is an endothelial cell mitogen and an important stimulator of sinusoidal endothelial cell proliferation.^[51]^ VEGF has a potent anti-apoptotic effect on hepatocytes through cell-cell interaction between sinusoidal endothelial cells and hepatocytes.^[52]^

The KEGG pathway enrichment analysis demonstrates a strong correlation between the mechanisms of SGF in mitigating DILI and various biological processes, including apoptosis, the TNF signaling pathway, and the PI3K-Akt signaling pathway. Hepatocellular apoptosis is present during the early phases of human liver failure.^[53]^ Apoptosis plays a critical role in the development of various liver diseases, such as cholestatic liver injury, alcoholic hepatopathy, diabetic hepatopathy, and drug-induced liver injury.^[54]^ The P53-upregulated modulator of apoptosis (PUMA) holds significant importance in mitigating liver injury induced by APAP. Additionally, Zhishi demonstrates hepatoprotective effects against APAP-induced liver necrosis by inhibiting PUMA.^[55,56]^ The TNF signaling pathway plays a crucial role in various physiological and pathological processes, including regulation of immune reactions, cell proliferation, apoptosis, and inflammation induction. Numerous drugs have the capability to enhance inflammation and cytokine release, rendering liver cells susceptible to the cytotoxic impacts of cytokines like TNF.^[57]^ pretreatment with American ginseng berry has been found to mitigate APAP-induced liver injury by suppressing oxidative stress and inflammation responses through the TNF- α -mediated signaling pathways.^[58]^ The PI3K/AKT pathway has been identified as a crucial player in liver regeneration post-injury. exerts its hepatoprotective effects by regulating the expression of downstream target proteins, either inhibiting or enhancing their expression.^[59,60]^ Research has demonstrated that leonurine effectively alleviates DILI by modulating the PI3K/ AKT signaling pathway.^[61]^

The molecular docking findings have provided additional evidence that the primary active constituents of SGF possess robust binding affinity towards crucial targets linked to drug-induced liver injury (DILI). Specifically, luteolin exhibits strong binding activity with ESR1, kaempferol with ESR1, and tanshinone iia with IL6, thus indicating their substantial binding capabilities. The findings from molecular docking analysis provide additional evidence supporting the potential of the chemical constituents found in SGF as promising therapeutic agents for DILI. Nevertheless, it is important to acknowledge the limitations inherent in this study. Considering the impact of diverse subjective and objective factors, it is crucial to undertake subsequent animal experiments and clinical trials to authenticate the predictive results.

It is important to acknowledge the limitations of this study. Firstly, the active compounds of SGF were obtained from literature and databases without experimental verification using LC/MS technology. Secondly, additional pharmacological experiments are required to validate the therapeutic mechanism of SGF on drug-induced liver injury (DILI).

## 5. Conclusion

By employing a combination of systematic pharmacology and molecular docking techniques, our research has provided preliminary insights into the target action pathway and molecular mechanism underlying the therapeutic effects of adding and modified SGF in the treatment of DILI. Our study findings have verified that SGF exhibits efficacy in treating DILI by engaging various active constituents, targeting multiple sites, and influencing diverse biological pathways. The primary active components identified in SGF include luteolin, kaempferol, and tanshinone iia, while the key targets implicated in its therapeutic action encompass IL6, ESR1, HIF1A, MYC, and VEGFA. Furthermore, it is plausible that SGF elicits its therapeutic effects via the initiation of apoptosis, adjustment of the TNF signaling pathway, and activation of the PI3K-Akt signaling pathway. In conclusion, our findings establish a theoretical framework for conducting in vitro and in vivo investigations, as well as clinical utilization of SGF in the management of DILI.

## Author contributions

**Conceptualization:** Ying Wang, Yang Ye.

**Data curation:** Xueying Chen, Yan Wang, Hong Zhong.

**Funding acquisition:** Ying Wang, Xueying Chen, Liqin Liu.

**Methodology:** Ying Wang, Xueying Chen, Yan Wang.

**Project administration:** Liqin Liu, Yang Ye.

**Writing – original draft:** Ying Wang, Hong Zhong.

**Writing – review & editing:** Xueying Chen, Yang Ye.

## References

[1] Kuna L Bozic I Kizivat T. Models of Drug Induced Liver Injury (DILI) - Current issues and future perspectives. Curr Drug Metab. 2018;19:830–8. 29788883 10.2174/1389200219666180523095355 PMC6174638

[2] Kullak-Ublick GA Andrade RJ Merz M. Drug-induced liver injury: recent advances in diagnosis and risk assessment. Gut. 2017;66:1154–64. 28341748 10.1136/gutjnl-2016-313369 PMC5532458

[3] Kumachev A Wu PE. Drug-induced liver injury. CMAJ. 2021;193:E310. 33649170 10.1503/cmaj.202026 PMC8034304

[4] Germani G Battistella S Ulinici D. Drug induced liver injury: from pathogenesis to liver transplantation. Minerva Gastroenterol. 2021;67:50–64. 10.23736/S2724-5985.20.02795-6 33222432

[5] European Association for the Study of the Liver. Electronic address: easloffice@easloffice.eu; Clinical Practice Guideline Panel: Chair; Panel members; EASL Governing Board representative. EASL clinical practice guidelines: drug-induced liver injury. J Hepatol. 2019;70:1222–61. 30926241 10.1016/j.jhep.2019.02.014

[6] Björnsson HK Björnsson ES. Drug-induced liver injury: pathogenesis, epidemiology, clinical features, and practical management. European J Internal Med. 2022;97:26–31. 34772600 10.1016/j.ejim.2021.10.035

[7] Hoofnagle JH Björnsson ES. Drug-induced liver injury - types and phenotypes. N Engl J Med. 2019;381:264–73. 31314970 10.1056/NEJMra1816149

[8] Zhou Y Wang C Kou J. Chrysanthemi Flos extract alleviated acetaminophen-induced rat liver injury via inhibiting oxidative stress and apoptosis based on network pharmacology analysis. Pharm Biol. 2021;59:1378–87. 34629029 10.1080/13880209.2021.1986077 PMC8510625

[9] Lv S Yu H Liu X. The study on the mechanism of Hugan tablets in treating drug-induced liver injury induced by atorvastatin. Front Pharmacol. 2021;12:683707. 34262454 10.3389/fphar.2021.683707 PMC8275032

[10] Li X Ge J Li M. Network pharmacology, molecular docking technology integrated with pharmacodynamic study to reveal the potential targets of Schisandrol A in drug-induced liver injury by acetaminophen. Bioorg Chem. 2022;118:105476. 34788696 10.1016/j.bioorg.2021.105476

[11] Liu YH Guo Y Feng H. Observation on the therapeutic effect of Shugan Jiedu decoction on anti-tuberculosis drug-induced liver injury. Chinese Integr Trad Western Med Liver Dis. 2020;30:453–4.

[12] Hopkins AL. Network pharmacology: the next paradigm in drug discovery. Nat Chem Biol. 2008;4:682–90. 18936753 10.1038/nchembio.118

[13] Li X Wei S Niu S. Network pharmacology prediction and molecular docking-based strategy to explore the potential mechanism of Huanglian Jiedu Decoction against sepsis. Comput Biol Med. 2022;144:105389. 35303581 10.1016/j.compbiomed.2022.105389

[14] Huang SJ Mu F Li F. Systematic elucidation of the potential mechanism of Erzhi Pill against drug-induced liver injury via network pharmacology approach. Evid Based Complement Altern Med. 2020;2020:6219432. 10.1155/2020/6219432 PMC6970004 31998398

[15] Kuang J Wu K Li W. Mechanism of Yangxinshi intervention on cardiac fibrosis in diabetic cardiomyopathy based on network pharmacology. Evid Based Complement Altern Med. 2022;2022:3968494. 10.1155/2022/3968494 PMC8799326 35096111

[16] Wang Y Yuan Y Wang W. Mechanisms underlying the therapeutic effects of Qingfeiyin in treating acute lung injury based on GEO datasets, network pharmacology and molecular docking. Comput Biol Med. 2022;145:105454. 35367781 10.1016/j.compbiomed.2022.105454

[17] Huang J Cheung F Tan HY. Identification of the active compounds and significant pathways of yinchenhao decoction based on network pharmacology. Mol Med Rep. 2017;16:4583–92. 28791364 10.3892/mmr.2017.7149 PMC5646998

[18] Liang T Kong Y Tang L. Network pharmacology-based analysis on the potential biological mechanisms of Yinzhihuang oral liquid in treating neonatal hyperbilirubinemia. Evid Based Complement Altern Med. 2022;2022:1672670. 10.1155/2022/1672670 PMC9556251 36248427

[19] Breuza L Poux S Estreicher A. The UniProtKB guide to the human proteome. Database. 2016;2016:bav120. 26896845 10.1093/database/bav120 PMC4761109

[20] Shannon P Markiel A Ozier O. Cytoscape: a software environment for integrated models of biomolecular interaction networks. Genome Res. 2003;13:2498–504. 14597658 10.1101/gr.1239303 PMC403769

[21] Barrett T Wilhite SE Ledoux P. NCBI GEO: archive for functional genomics data sets—update. Nucleic Acids Res. 2013;41:D991–995. 23193258 10.1093/nar/gks1193 PMC3531084

[22] Rebhan M Chalifa-Caspi V Prilusky J. GeneCards: integrating information about genes, proteins and diseases. Trends Genet. 1997;13:163. 9097728 10.1016/s0168-9525(97)01103-7

[23] Amberger JS Bocchini CA Schiettecatte F. OMIMorg: Online Mendelian Inheritance in Man (OMIM®), an online catalog of human genes and genetic disorders. Nucleic Acids Res. 2015;43:D789–798. 25428349 10.1093/nar/gku1205 PMC4383985

[24] Sayers EW Beck J Bolton EE. Database resources of the national center for biotechnology information. Nucleic Acids Res. 2021;49:D10–7. 33095870 10.1093/nar/gkaa892 PMC7778943

[25] Piñero J Ramírez-Anguita JM Saüch-Pitarch J. The DisGeNET knowledge platform for disease genomics: 2019 update. Nucleic Acids Res. 2020;48:D845–55. 31680165 10.1093/nar/gkz1021 PMC7145631

[26] Szklarczyk D Franceschini A Wyder S. STRING v10: protein-protein interaction networks, integrated over the tree of life. Nucleic Acids Res. 2015;43:D447–452. 25352553 10.1093/nar/gku1003 PMC4383874

[27] Chin CH Chen SH Wu HH. cytoHubba: identifying hub objects and sub-networks from complex interactome. BMC Syst Biol. 2014;8(Suppl 4):S11. 25521941 10.1186/1752-0509-8-S4-S11 PMC4290687

[28] Guo A Wang W Shi H. Identification of hub genes and pathways in a rat model of renal ischemia-reperfusion injury using bioinformatics analysis of the gene expression omnibus (GEO) dataset and integration of gene expression profiles. Med Sci Monitor. 2019;25:8403–11. 10.12659/MSM.920364 PMC6863034 31699960

[29] Song W Ni S Fu Y. Uncovering the mechanism of Maxing Ganshi Decoction on asthma from a systematic perspective: a network pharmacology study. Sci Rep. 2018;8:17362. 30478434 10.1038/s41598-018-35791-9 PMC6255815

[30] DennisGYangJGaoW. DAVID: database for annotation, visualization, and integrated discovery. Genome Biol. 2003;4:P3.12734009

[31] Trott O Olson AJ. AutoDock Vina: improving the speed and accuracy of docking with a new scoring function, efficient optimization, and multithreading. J Comput Chem. 2010;31:455–61. 19499576 10.1002/jcc.21334 PMC3041641

[32] Kim S Chen J Cheng T. PubChem in 2021: new data content and improved web interfaces. Nucleic Acids Res. 2021;49:D1388–95. 33151290 10.1093/nar/gkaa971 PMC7778930

[33] Berman HM Westbrook J Feng Z. The protein data bank. Nucleic Acids Res. 2000;28:235–42. 10592235 10.1093/nar/28.1.235 PMC102472

[34] Patel SJ Milwid JM King KR. Gap junction inhibition prevents drug-induced liver toxicity and fulminant hepatic failure. Nat Biotechnol. 2012;30:179–83. 22252509 10.1038/nbt.2089 PMC3609650

[35] Tujios S Fontana RJ. Mechanisms of drug-induced liver injury: from bedside to bench. Nature Rev Gastroenterol Hepatol. 2011;8:202–11. 21386809 10.1038/nrgastro.2011.22

[36] Weaver RJ Blomme EA Chadwick AE. Managing the challenge of drug-induced liver injury: a roadmap for the development and deployment of preclinical predictive models. Nat Rev Drug Discov. 2020;19:131–48. 31748707 10.1038/s41573-019-0048-x

[37] Jiang Z Han Y Zhang Y. Sedum sarmentosum bunge attenuates drug-induced liver injury via Nrf2 signaling pathway: an experimental verification based on network pharmacology prediction. J Healthcare Eng. 2021;2021:1142638. 10.1155/2021/1142638 PMC8577938 34900173

[38] Shen Z Yu M Zhang S. Network pharmacology and molecular docking analyses unveil the mechanisms of Yiguanjian Decoction against Parkinson’s Disease from inner/outer brain perspective. Biomed Res Int. 2022;2022:4758189. 36237735 10.1155/2022/4758189 PMC9552692

[39] Liu S Tian L Chai G. Targeting heme oxygenase-1 by quercetin ameliorates alcohol-induced acute liver injury via inhibiting NLRP3 inflammasome activation. Food Function. 20148;9:4184–93. 10.1039/c8fo00650d 29993075

[40] Wang H Chen L Zhang X. Kaempferol protects mice from d-GalN/LPS-induced acute liver failure by regulating the ER stress-Grp78-CHOP signaling pathway. Biomed Pharmacother. 2019;111:468–75. 30594786 10.1016/j.biopha.2018.12.105

[41] Tai M Zhang J Song S. Protective effects of luteolin against acetaminophen-induced acute liver failure in mouse. Int Immunopharmacol. 2015;27:164–70. 26002582 10.1016/j.intimp.2015.05.009

[42] Wang W Guan C Sun X. Tanshinone IIA protects against acetaminophen-induced hepatotoxicity via activating the Nrf2 pathway. Phytomedicine. 2016;23:589–96. 27161400 10.1016/j.phymed.2016.02.022

[43] Rousta AM Mirahmadi SM Shahmohammadi A. Therapeutic potential of isorhamnetin following acetaminophen-induced hepatotoxicity through targeting NLRP3/NF-κB/Nrf2. Drug Res. 2022;72:245–54. 10.1055/a-1792-2678 35359022

[44] Michalopoulos GK. Liver regeneration. J Cell Physiol. 2007;213:286–300. 17559071 10.1002/jcp.21172 PMC2701258

[45] Gao RY Wang M Liu Q. Hypoxia-inducible factor-2α reprograms liver macrophages to protect against acute liver injury through the production of Interleukin-6. Hepatology (Baltimore, Md.). 2020;71:2105–17. 10.1002/hep.30954 PMC7075728 31529728

[46] Taub R. Hepatoprotection via the IL-6/Stat3 pathway. J Clin Invest. 2003;112:978–80. 14523032 10.1172/JCI19974 PMC198534

[47] Yan Z Tan W Dan Y. Estrogen receptor alpha gene polymorphisms and risk of HBV-related acute liver failure in the Chinese population. BMC Med Genet. 2012;13:49. 22727021 10.1186/1471-2350-13-49 PMC3412699

[48] Giannitrapani L Soresi M La Spada E. Sex hormones and risk of liver tumor. Ann N Y Acad Sci. 2006;1089:228–36. 17261770 10.1196/annals.1386.044

[49] Lee KA Roth RA LaPres JJ. Hypoxia, drug therapy and toxicity. Pharmacol Ther. 2007;113:229–46. 17046066 10.1016/j.pharmthera.2006.08.001

[50] Sparkenbaugh EM Saini Y Greenwood KK. The role of hypoxia-inducible factor-1α in acetaminophen hepatotoxicity. J Pharmacol Exp Ther. 2011;338:492–502. 21576378 10.1124/jpet.111.180521 PMC3141908

[51] Marino G Piazzese E Gruttadauria S. [Innovative use of the vascular endothelial growth factor in an experimental model of acute liver failure]. Il Giornale di chirurgia. 2004;25:61–4. 15219100

[52] Tanaka Y Sohda T Matsuo K. Vascular endothelial growth factor reduces Fas-mediated acute liver injury in mice. J Gastroenterol Hepatol. 2008;23:e207–211. 17784864 10.1111/j.1440-1746.2007.05135.x

[53] Possamai LA McPhail MJ Quaglia A. Character and temporal evolution of apoptosis in acetaminophen-induced acute liver failure*. Crit Care Med. 2013;41:2543–50. 23949472 10.1097/CCM.0b013e31829791a2 PMC3939768

[54] Chen SN Tan Y Xiao XC. Deletion of TLR4 attenuates lipopolysaccharide-induced acute liver injury by inhibiting inflammation and apoptosis. Acta Pharmacol Sin. 2021;42:1610–9. 33495514 10.1038/s41401-020-00597-x PMC8463538

[55] Shu Y He D Li W. Hepatoprotective effect of Citrus aurantium L. Against APAP-induced liver injury by regulating liver lipid metabolism and apoptosis. Int J Biol Sci. 2020;16:752–65. 32071546 10.7150/ijbs.40612 PMC7019131

[56] Iorga A Dara L Kaplowitz N. Drug-induced liver injury: cascade of events leading to cell death, apoptosis or necrosis. Int J Mol Sci . 2017;18:1018. 28486401 10.3390/ijms18051018 PMC5454931

[57] Han D Shinohara M Ybanez MD. Signal transduction pathways involved in drug-induced liver injury. Handb Exp Pharmacol. 2010:267–310. 10.1007/978-3-642-00663-0_10 20020266

[58] Xu XY Wang Z Ren S. Improved protective effects of American ginseng berry against acetaminophen-induced liver toxicity through TNF-α-mediated caspase-3/-8/-9 signaling pathways. Phytomedicine. 2018;51:128–38. 30466610 10.1016/j.phymed.2018.09.234

[59] Leng J Wang Z Fu CL. NF-κB and AMPK/PI3K/Akt signaling pathways are involved in the protective effects of Platycodon grandiflorum saponins against acetaminophen-induced acute hepatotoxicity in mice. Phytother Res. 2018;32:2235–46. 30039882 10.1002/ptr.6160

[60] Li Y Lu L Luo N. Inhibition of PI3K/AKt/mTOR signaling pathway protects against d-galactosamine/lipopolysaccharide-induced acute liver failure by chaperone-mediated autophagy in rats. Biomed Pharmacother. 2017;92:544–53. 28577493 10.1016/j.biopha.2017.05.037

[61] Yu Y Zhou S Wang Y. Leonurine alleviates acetaminophen-induced acute liver injury by regulating the PI3K/AKT signaling pathway in mice. Int Immunopharmacol. 2023;120:110375. 37267857 10.1016/j.intimp.2023.110375

